# Inflammatory prognostic scoring systems are risk factors for surgical site infection following wide local excision of soft tissue sarcoma

**DOI:** 10.1007/s00590-021-03142-6

**Published:** 2021-10-09

**Authors:** Omer M. Farhan-Alanie, Taegyeong Tina Ha, James Doonan, Ashish Mahendra, Sanjay Gupta

**Affiliations:** Department of Musculoskeletal Oncology Surgery, Glasgow Royal Infirmary, 84 Castle St, Glasgow, G4 0SF United Kingdom

**Keywords:** Soft tissue sarcoma, Infection, Inflammatory prognostic score, Modified Glasgow prognostic score

## Abstract

**Introduction:**

Limb-sparing surgery with negative margins is possible in most soft tissue sarcoma (STS) resections and focuses on maximising function and minimising morbidity. Various risk factors for surgical site infections (SSIs) have been reported in the literature specific to sarcoma surgery. The aim of this study is to determine whether systemic inflammatory response prognostic scoring systems can predict post-operative SSI in patients undergoing potentially curative resection of STS.

**Methods:**

Patients who had a planned curative resection of a primary STS at a single centre between January 2010 and December 2019 with a minimum follow-up of 6 months were included. Data were extracted on patient and tumour characteristics, and pre-operative blood results were used to calculate inflammatory prognostic scores based on published thresholds and correlated with risk of developing SSI or debridement procedures.

**Results:**

A total of 187 cases were included. There were 60 SSIs. On univariate analysis, there was a statistically significant increased risk of SSI in patients who are diabetic, increasing specimen diameter, American Society of Anaesthesiology (ASA) grade 3, use of endoprosthetic replacement, blood loss greater than 1 L, and junctional tumour location. Modified Glasgow prognostic score, C-reactive protein/albumin ratio and neutrophil–platelet score (NPS) were statistically associated with the risk of SSI. On multivariate analysis, ASA grade 3, junctional tumour location and NPS were independently associated with the risk of developing a SSI.

**Conclusion:**

This study supports the routine use of simple inflammation-based prognostic scores in identifying patients at increased risk of developing infectious complications in patients undergoing potentially curative resection of STS.

## Introduction

The principles of management of soft tissue sarcoma (STS) have evolved considerably in recent years. Limb-sparing surgery with negative margins is possible in the majority of resections and focuses on maximising function and minimising morbidity [1, 2]. Amputation is only required in approximately 10% of cases, where the involvement of critical anatomic structures prevents a curative wide local surgical resection [3]. However, limb-sparing resections often involve large surgical fields, and surgical site infection (SSI) remains an important source of post-operative morbidity [4]. Wound complications including dehiscence, cellulitis, abscess, seromas, haematomas and wound necrosis have been reported to occur in 16–56% cases in the published literature [4, 5].

Various risk factors for surgical site infections have been reported in the literature specific to sarcoma surgery, and these can be sub-classified into surgical factors, host factors and adjunctive treatment factors [5–7]. Large tumour resections, particularly involving the adductor compartment of the thigh, use of adjuvant radiotherapy, and a variety of patient-specific factors including smoking, diabetes and obesity, have all been reported to independently affect the risk of SSIs [8]. Identification and knowledge of such risk factors is important as it allows appropriate patient counselling and can guide future research in reducing these risks. Recent proposals have focussed on these high risk wounds and look to utilise existing technology such as negative pressure wound therapy to mitigate these risk factors [9]. There is also evidence to support the immediate use of free-flap reconstruction, particularly in patients who receive neoadjuvant radiotherapy, in an effort to substitute irradiated soft tissue for healthy soft tissue from the donor site, and optimising the vascularisation of the resection site [10].

There has been increasing evidence over the last two decades that the host response to malignancy plays a key role in the prognosis and outcomes of treatment. A systemic inflammatory response is a proposed unifying model for this host interaction with the tumour, with various scoring systems proposed to quantify this [11–14]. These scoring systems rely on routine haematological and biochemical laboratory parameters to stratify this response, and they have been shown in numerous studies to correlate with survival in a variety of malignancies [13, 15]. More recent work has focussed on their use in predicting post-operative morbidity, particularly in potentially curative carcinoma resections [16].

However, only limited data are available on the applicability of systemic inflammatory response prognostic scores in the management of soft tissue sarcomas, which represent a different cellular lineage to carcinomas [17]. Emerging evidence supports a correlation between the modified Glasgow prognostic score (mGPS) and survival in soft tissue sarcoma, but no studies have investigated their relationship with post-operative SSIs in soft tissue sarcoma. Therefore, the aim of this study is to determine whether systemic inflammatory response prognostic scoring systems can independently predict post-operative surgical site infection in patients undergoing potentially curative resection of soft tissue sarcoma.

## Methods

All patients who had a planned curative resection of a primary STS in the West of Scotland between the calendar years January 2010 and December 2019 with a minimum follow-up of 6 months were entered into the study. These patients were identified from a prospectively maintained database. Review of the electronic case notes was performed for 213 patients who met the inclusion criteria. Any patient with metastatic disease at presentation, a low grade tumour subtype (atypical lipomatous tumour/dermatofibrosarcoma protuberans) or incomplete data were excluded to avoid selection and transfer bias. As a result of access to a national electronic case note files, no patients were lost to follow-up.

All patients were treated surgically under the supervision of the two senior authors (AM and SG). Data were extracted on sex, age, height, weight, use of adjuvant therapy, co-morbidities, American Society of Anaesthesiology (ASA) grade, intra-operative blood loss, tumour size, tumour grade, specimen size, location of tumour and need for immediate soft tissue reconstruction. We grouped tumour location into anatomic location but described a subset which we defined as junctional, which occur in the axilla, groin, popliteal fossa and distal medial thigh. All patients received a low-pressure vacuum drain in the immediate post-operative period, which was left in situ until drainage was less than 50 ml over a 24-h period. Peri-operative antibiotic prophylaxis was routinely continued until drain removal. Thrombo-embolic prophylaxis consisted of low-molecular weight heparin, which was maintained as inpatient therapy for all patients, and for 4 weeks post-operatively in lower limb sarcoma surgery.

Pre-operative blood results including C-reactive protein, albumin, white cell count, neutrophil, lymphocyte and platelet count were collected. These data were used to calculate established inflammatory scoring systems based on validated thresholds as demonstrated in Table 1 [13].

**Table 1:** Systemic inflammation-based prognostic ratios and scores

Ratio/score	Ratio/score
*NLR*	
Neutrophil count/lymphocyte count	≤ 3
Neutrophil count/lymphocyte count	3–5
Neutrophil count/lymphocyte count	> 5
*NLS*	
Neutrophil count ≤ 7.5 × 10^9^/l and lymphocyte count ≥ 1.5 × 10^9^/l	0
Neutrophil count > 7.5 × 10^9^/l and lymphocyte count ≥ 1.5 × 10^9^/l	1
Neutrophil count ≤ 7.5 × 10^9^/l and lymphocyte count < 1.5 × 10^9^/l	1
Neutrophil count > 7.5 × 10^9^/l and lymphocyte count < 1.5 × 10^9^/l	2
*PLR*	
Platelet count/lymphocyte count	≤ 150
Platelet count/lymphocyte count	> 150
*PLS*	
Platelet count ≤ 400 × 10^9^/l and lymphocyte count ≥ 1.5 × 10^9^/l	0
Platelet count > 400 × 10^9^/l and lymphocyte count ≥ 1.5 × 10^9^/l	1
Platelet count ≤ 400 × 10^9^/l and lymphocyte count < 1.5 × 10^9^/l	1
Platelet count > 400 × 10^9^/l and lymphocyte count < 1.5 × 10^9^/l	2
*LMR*	
Lymphocyte count/monocyte count	≥ 2.40
Lymphocyte count/monocyte count	< 2.40
*LMS*	
Lymphocyte count ≥ 1.5 × 10^9^/l and monocyte count ≤ 0.8 × 10^9^/l	0
Lymphocyte count ≥ 1.5 × 10^9^/l and monocyte count ≤ 0.8 × 10^9^/l	1
Lymphocyte count < 1.5 × 10^9^/l and monocyte count > 0.8 × 10^9^/l	1
Lymphocyte count < 1.5 × 10^9^/l and monocyte count > 0.8 × 10^9^/l	2
*NPS*	
Neutrophil count ≤ 7.5 × 10^9^/l and platelet count < 400 × 10^9^/l	0
Neutrophil count > 7.5 × 10^9^/l and platelet count < 400 × 10^9^/l	1
Neutrophil count ≤ 7.5 × 10^9^/l and platelet count > 400 × 10^9^/l	1
Neutrophil count > 7.5 × 10^9^/l and platelet count > 400 × 10^9^/l	2
*CAR*	
C-reactive protein/albumin	≤ 0.22
C-reactive protein/albumin	> 0.22
*mGPS*	
C-reactive protein ≤ 10 mg/l and albumin ≥ 35 g/l	0
C-reactive protein > 10 mg/l and albumin ≥ 35 g/l	1
C-reactive protein > 10 mg/l and albumin < 35 g/l	2

*NLR* Neutrophil–lymphocyte ratio; *NLS* neutrophil–lymphocyte score; *PLR* platelet–lymphocyte ratio; *PLS* platelet–lymphocyte score; *LMR* lymphocyte–monocyte ratio; *LMS* lymphocyte–monocyte score; *NPS* neutrophil–platelet score; *CAR* C-reactive protein–albumin ratio; *mGPS* modified Glasgow prognostic score

Data were collected on post-operative surgical site infections (SSIs). Patients were routinely followed up at 2 weeks post-operatively at the sarcoma clinic, then at regular intervals thereafter until the wound is satisfactory. If patients underwent soft tissue reconstruction, then the immediate post-operative follow-up was by the onco-plastic team weekly for the first 4 weeks. If post-operative radiotherapy was recommended, this was instituted once the wound was deemed satisfactory by the surgical team, and standard treatment was to commence this at 6 weeks post-operatively. We defined a post-operative SSI as a surgical site requiring treatment with antibiotic therapy or an infective complication that required surgical intervention such as debridement and washout of the surgical site. No ethical approval was required for this study.

## Statistics

Variables were groups by standard binary or categorical thresholds. Univariate survival analysis was performed using a Cox proportional hazards model taking into account time to surgical site infection. Kaplan–Meir analysis using log-rank test was used to graphically demonstrate significance. A *p*-value of < 0.05 was considered statistically significant. Multivariate analysis was performed using a Cox proportional hazards model with a stepwise backward procedure to derive a final model of the variables that had a significant independent relationship with post-operative surgical site complications.

Inter-relationships between variables were assessed using contingency table analysis with the Chi square test for trend as appropriate. For variables with few observations, Fisher exact test was used. Analysis was performed using SPSS software (version 26.0.0. SPSS Inc, Chicago, IL, USA) or GraphPad Prism (version 6, San Diego, CA, USA).

## Results

Between January 2010 and December 2019, there were 187 cases eligible for analysis. A summary of the clinico-pathological characteristics of the patients is presented in Table 2. There were 60 surgical site infections; 21 of these were treated with antibiotic therapy, and 39 patients required an additional surgical procedure. Median time to diagnosis of any surgical site infection was 22 days (interquartile range 14–40 days), and median time to surgical intervention was 21 days (interquartile range 16–34 days). There were 21 distinct subtypes of soft tissue sarcomas within our group, and these are summarised in Table 3.

**Table 2:** Variable distribution and univariate Cox regression analysis

Variable	Total	No complication	Complication	*p*-value	Hazard ratio (95% CI)
Number of patients	187	127	60	–	
Sex				0.938	
Male	90	61	29		
Female	97	66	31		
Mean age in years (Range)	59.0 (19–93)	59 (20–91)	59 (19–93)	0.960	
Mean BMI (range)	28.3 (17.7–68.5)	27.9 (17.7–68.5)	29.1 (19.4–55.5)	0.219	
Trojani tumour grade				0.175	
Grade 1	16	13	3		
Grade 2	49	35	14		
Grade 3	122	79	43		
Mean tumour diameter in cm (range)	9.42 ( 0.5–31.0)	9.0 (0.5 – 31.0)	10.3 (1.3–27.5)	0.206	
Mean specimen diameter in cm (range)	16.1 (2.0–39.0)	14.9 (2.0–39.0)	18.7 (3.0–34.0)	**0.003**	1.06 (1.02–1.09)
Tumour site					4.69 (1.11–19.76)
Trunk	15	13	2	–	
Upper limb	35	29	6	0.73	
Lower limb	86	60	26	0.218	
Junctional	51	25	26	0.035	
Adjuvant radiotherapy					
None	57	42	15	–	
Adjuvant	98	66	32	0.615	
Neoadjuvant	32	19	13	0.217	
Wound closure				0.486	
Primary	95	67	28		
Soft tissue reconstruction	92	60	32		
ASA grade					
II	143	105	38	–	2.42 (1.42–4.12)
III	39	18	21	**0.001** 0.812	
IV	5	4	1		
Diabetes mellitus				**0.045**	1.95 (1.01–3.76)
Yes	165	116	11		
No	22	11	49		
Hypertension				0.147	
Yes	118	15	8		
No	68	112	51		
Hypercholesterolaemia	163	15	8	0.819	
Yes	23	112	51		
No					
Smoker				0.744	
Active	30	20	10		
Non-active	157	107	50		
Surgery type				0.646	
Ablative	26	19	7		
Limb sparing	161	108	53		
Bone reconstruction				**0.028**	2.43 (1.10–5.35)
Nil	177	124	53		
Endoprosthesis	10	3	7		
Blood loss				**0.022**	2.22 (1.12–4.38)
< 1 Litre	170	120	50		
> 1 Litre	17	7	10		
mGPS				**0.019**	1.41 (1.06–1.88)
0	108	81	27		
1	38	24	14		
2	41	22	19		
CAR				**0.04** **8**	1.67 (1.004–2.79)
0	100	74	26		
1	84	50	34		
NLR				0.182	
0	89	65	24		
1	58	37	12		
2	38	23	15		
NLS				0.230	
0	99	70	29		
1	78	51	27		
2	8	4	4		
PLR				0.103	
0	60	46	14		
1	125	79	46		
PLS				0.131	
0	94	67	27		
1	84	56	28		
2	7	2	5		
NPS				**0.004**	1.73 (1.19–2.52)
0	148	107	41		
1	28	15	13		
2	9	3	6		

BMI = body mass index, CI = confidence interval

Bold represents statistically significant finding

**Table 3:** Tumour histology types included in study

Tumour type	*n*
Undifferentiated sarcoma NOS	34
Undifferentiated pleomorphic sarcoma	25
Myxoid liposarcoma	24
Spindle cell sarcoma	24
Myxofibrosarcoma	18
Leiomyosarcoma	17
Synovial sarcoma	10
Liposarcoma	7
Rhabdomyosarcoma	5
Malignant peripheral nerve sheath	5
Extraskeletal osteosarcoma	3
Fibromyxoid sarcoma	3
Angiosarcoma	3
Myofibroblastic sarcoma	2
Pleomorphic hyalinising angiectatic	1
Epithelioid sarcoma	1
Small cell neuroendocrine	1
Clear cell sarcoma	1
Fibromyxoid sarcoma	1
Myxoinflammatory fibroblastic sarcoma	1
Round cell sarcoma	1

NOS: not otherwise specified

Univariate analysis determined that there was a statistically significant increased risk of SSI in patients who are diabetic (50% versus 29.7%, *p* = 0.045) and patients with an ASA grade 3 (53.8% versus 26.6% ASA 2 patients, *p* = 0.001). There was no significant association between other patient related risk factors such as sex, age, smoking status, Body Mass Index (BMI), hypertension or hypercholesterolaemia.

With regard to pathological characteristics, the mean tumour diameter was 9.42 cm and the mean specimen diameter was 16.1 cm. The majority of tumours were Trojani grade 3 (65.2%). There was a statistically significant 6% increased risk of complications per centimetre increase in the maximum measured specimen diameter (*p* = 0.003). There was no association between risk of complication and maximum measured tumour diameter or Trojani tumour grade.

Surgical factors associated with increased risk of post-operative SSI include intra-operative blood loss greater than 1 L (58.8% versus 29.4%, *p* = 0.022), junctional location of tumour (51% versus 17.1% in upper limb, *p* = 0.035) and implantation of an endoprosthesis (70% versus 29.9%, *p* = 0.028). There was no statistically significant association with need for primary amputation, neoadjuvant or adjuvant radiotherapy or type of wound closure.

We examined the association of SSI with various previously described systemic inflammatory scoring systems. There was a statistically significant association between the modified Glasgow prognostic scoring system (mGPS), C-reactive protein-to-albumin ratio (CAR), neutrophil–platelet score (NPS) and risk of developing a surgical site infection (all *p* < 0.05). There was no statistically significant association on univariate analysis with the neutrophil–lymphocyte ratio (NLR), neutrophil–lymphocyte score (NLS), platelet–lymphocyte ratio (NLR) and platelet–lymphocyte score (PLS) and the risk of developing post-operative SSI.

**Fig. 1:**
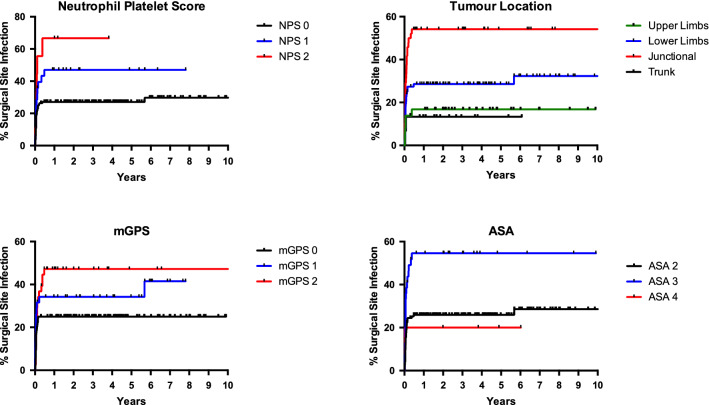
Kaplan–Meir survival graph demonstrating risk of surgical site infection with a) NPS, b) mGPS, c) tumour location and d) ASA grade

On multivariate analysis, we identified three independent risk factors for developing a post-operative SSI (Table 4). The NPS was significantly and independently associated with an increased risk of SSI, with a hazard ratio of 1.64 (95% CI 1.11–2.40) per level increase in value (*p* = 0.012). Additionally, ASA grade 3 was associated with a hazard ratio of 2.24 (1.30–3.85) when compared to ASA grade 2 (*p* = 0.004), and a tumour located in a junctional area was associated with a hazard ratio of 3.74 (1.53–9.12) for developing a SSI (*p* = 0.004). Figure 1 demonstrates the Kaplan–Meir survival curves for these factors.

**Table 4:** Statistical association between variables included in study and systemic inflammatory scoring systems significant on univariate analysis

Variable	NPS	CAR	mGPS
Sex	0.333	0.685	0.584
Age	**0.034**	** < 0.001**	**0.002**
BMI	0.710	0.083	0.106
Trojani tumour grade	**0.016**	** < 0.001**	** < 0.001**
Tumour diameter	**0.001**	** < 0.001**	** < 0.001**
Specimen diameter	**0.004**	** < 0.001**	** < 0.001**
Tumour site	0.199	0.313	0.245
Adjuvant radiotherapy	0.103	**0.014**	0.054
Wound closure	0.478	**0.008**	0.054
ASA grade	**0.041**	0.399	**0.002**
Diabetes mellitus	0.901	0.663	0.818
Hypertension	0.572	0.392	0.179
Hypercholesterolaemia	0.079	0.519	0.788
Smoker	0.916	0.060	0.352
Surgery type	0.215	**0.029**	**0.027**
Bone reconstruction	0.161	0.516	0.393
Blood loss	**0.001**	**0.040**	** < 0.001**

Bold represents statistically significant finding

We examined associations between the variables collected and the three systemic inflammatory scoring systems that were statistically significant on univariate analysis, namely mGPS, CAR and NPS (Table 5). There was a statistically significant association of all three scoring systems with increasing age, higher-grade tumour, increased tumour diameter size and specimen size, and increased intra-operative blood loss. NPS and mGPS were both statistically significantly associated with increased ASA score. Patients with an elevated mGPS and CAR were more likely to undergo ablative surgery. Patients with an elevated CAR were statistically significantly associated with an increased rate of soft tissue reconstruction.

**Table 5:** Variables determined to predict surgical site infection on multivariate analysis

Variable	*P*-value	Hazard ratio (95% CI)
ASA grade 3	0.004	2.24 (1.29–3.88)
Neutrophil–platelet score	0.012	1.64 (1.11–2.40)
Junctional tumour location	0.004	3.74 (1.53–9.12)

## Discussion

This study has demonstrated that the pre-operative systemic inflammatory prognostic scores, namely the modified Glasgow prognostic score, the C-reactive protein-to-albumin ratio and the neutrophil–platelet score, were all statistically significant prognostic factors for development of SSI in patients undergoing potentially curative resection of soft tissue sarcoma, with the latter also being an independent risk factor on multivariate analysis.

Post-operative SSI is relatively common in patients undergoing resection of soft tissue sarcoma, associated with increased hospital stay, treatment costs and may result in delays to adjuvant radiotherapy. Therefore, identifying at-risk patients is key to informed consent, but can also allow targeted interventions to mitigate these risks. The results of this study suggest that patients with an elevated NPS, ASA grade 3 or tumours resected from a junctional location may benefit from such interventions. Incisional negative pressure wound therapy has been utilised in orthopaedic and non-orthopaedic surgical scenarios, both routinely and targeted in at-risk wounds and has been shown to minimise the risk of post-operative wound complications, with an ongoing randomised control trial investigating its utility in soft tissue sarcoma surgery [18–21]. In addition, it has been shown that the use of soft tissue reconstruction can minimise the risk of surgical site infections in soft tissue sarcoma surgery, particularly patients who undergo neoadjuvant radiotherapy [22, 23]. Whilst our study found no association between the use soft tissue reconstruction and risk of complications, it is likely that the retrospective nature of this study resulted in a treatment selection bias. Indeed, we noted a higher than published rate of soft tissue reconstruction in our study cohort (49.1%), and this likely represents our evolution in practice with time to a more aggressive surgical approach using soft tissue reconstruction.

The basis of the independent relationship between an elevated systemic inflammatory response pre-operatively and post-operative infections in patients with primary operable soft tissue sarcoma is not clear. Whilst both the cell-mediated immune response and humoral immune response are associated with post-operative SSI, only the former was independently associated with complications. This is in contrast with disease-free survival in soft tissue sarcoma, which is more closely associated with the humoral immune response as measured by the mGPS [17]. It is also interesting to note that whilst the tumour size and grade were associated with an elevated mGPS, CAR and NPS, they were not associated with an increased risk of complications. We therefore hypothesise that whilst the tumour elicits a significant systemic inflammatory response in the host, it is this dysfunctional response that predisposes to the increased risk of SSI, and that these biomarkers measure the dysfunctional response rather than act as surrogates to tumour aggressiveness in the aetiology and prediction of surgical site infections.

Systemic inflammation and nutritional status are clearly inter-linked. Inflammation impairs nutritional status by reducing food intake and impairing micronutrient absorption, and malnutrition increases the risk and severity of inflammation [24, 25]. Whilst BMI was not associated with the risk of SSI or with an increased systemic inflammatory prognostic scores, it is well recognised that absolute BMI is a poor marker of nutritional status in cancer [26, 27]. Several studies have identified a link between inflammatory prognostic scores, skeletal muscle mass and cancer cachexia, and poor nutritional status has been demonstrated to increase the risk of peri-operative complications including surgical site infections [28–30]. Further research aimed at identifying a link between inflammatory prognostic scores, cancer cachexia and surgical site infection in soft tissue sarcoma can provide easy identification of these patients and potentiate a pathway for intervening in this group to potentially reduce their risk of SSI.

It is interesting to note that the mean specimen diameter was significantly associated with risk of post-operative surgical site infections, but that tumour diameter is not. To the best of our knowledge, this is the first paper to distinguish between tumour size and specimen size in the same cohort. This supports the hypothesis that it is the residual dead space created by resection of larger tumours which predisposes to development of seromas and hematomas, as well as subsequent infection [4]. The significant association between increased blood loss and risk of post-operative SSI is likely driven by a similar mechanism. It has also been suggested that intra-operative blood transfusion may act synergistically with surgical stress to induce immunosuppression, and increased blood loss is likely to be associated with higher risk of intra-operative blood transfusion [31, 32].

Anatomic location of tumour is also a significant prognostic indicator of SSI [2, 8, 33]. Prior studies have focussed on anatomic divisions, but we hypothesised that grouping patients based on this feature alone can result in a dilutional effect. We have shown that these at risk areas, namely the medial thigh, popliteal fossa, groin and axilla are more appropriately grouped and are an independent risk factors for SSI. It has been postulated that certain sites are more at risk of SSI due to en bloc resection of venous and lymphatic vessels, which result in seroma and subsequent infection [8, 33]. In addition, we propose two other features that may account for the high risk nature of these areas. There is likely a mechanical shear and tensile effect on the soft tissue in these areas, which often fall in junctional zones. In addition, these areas show a high concentration of gram negative or anaerobic flora, which may represent more opportunistic and more virulent micro-organisms [34].

We recognise that this is a heterogenous group of patients, in terms of both tumour aetiology and patient characteristics. This represents a limitation of our paper but is reflective of standard sarcoma surgeon’s practice. In addition, the heterogenous nature represents a further demand for a simplified, unified method of appropriately stratifying risk of surgical site infection in this varied cohort of patients, presentations and management types.

In summary, this study supports the routine use of simple inflammation-based prognostic scores in identifying patients at increased risk of developing infectious complications in patients undergoing potentially curative resection of soft tissue sarcoma. However, it remains to be determined whether the pre-operative systemic inflammatory response may be moderated and whether such moderation may reduce post-operative infectious complications, and further prospective work is required to clarify whether interventions can be targeted in this at risk group to minimise these complications.
